# Organic Thin Film Transistors Incorporating Solution Processable Thieno[3,2-*b*]thiophene Thienoacenes

**DOI:** 10.3390/ma11010008

**Published:** 2017-12-22

**Authors:** Nicole A. Rice, François Magnan, Owen A. Melville, Jaclyn L. Brusso, Benoît H. Lessard

**Affiliations:** 1 Department of Chemical and Biological Engineering, University of Ottawa, 161 Louis Pasteur, Ottawa, ON K1N 6N5, Canada; nrice@uottawa.ca (N.A.R.); omelv065@uottawa.ca (O.A.M.); 2 Department of Chemistry and Biomolecular Science, University of Ottawa, 10 Marie Curie, Ottawa, ON K1N 6N5, Canada; frank.magnan@gmail.com

**Keywords:** thieno[3,2-*b*]thiophene, organic thin film transistors, interface engineering, solution processing

## Abstract

Bottom-gate bottom-contact organic thin film transistors (OTFTs) were prepared with four novel star-shaped conjugated molecules containing a fused thieno[3,2-*b*]thiophene moiety incorporated either in the core and/or at the periphery of the molecular framework. The molecules were soluble in CS_2_, allowing for solution-processing techniques to be employed. OTFTs with different channel geometries were characterized in both air and vacuum in order to compare environmental effects on performance. Blending the small molecules with poly(styrene), an insulating polymer, facilitated the formation of an even semiconducting film, resulting in an order of magnitude increase in device mobility. The highest field-effect mobilities were in air and on the order of 10^−3^ cm^2^/Vs for three of the four molecules, with a maximum mobility of 9.2 × 10^−3^ cm^2^/Vs achieved for the most conjugated small molecule. This study explores the relationship between processing conditions and OTFT devices performance for four different molecules within this new family of materials, resulting in a deeper insight into their potential as solution-processable semiconductors.

## 1. Introduction

Organic thin film transistors (OTFTs) are fundamental components of organic electronic devices, such as flexible large-area displays, radio frequency identification tags, and integrated circuits [1,2,3,4,5]. Incorporation of organic semiconductors as active materials in electronics allows for the use of facile solution-based fabrication techniques [6]. This has the potential to decrease device fabrication costs by facilitating high-throughput manufacturing techniques. OTFTs also enable the production of electronic devices with increased mechanical flexibility through integration onto substrates with various rigidities. Finally, versatile chemical synthetic methodologies allows for the engineering of a plethora of soluble organic semiconductors, in which both the electronic properties and nanoscale morphology can be tuned.

Designing organic semiconductors with performance and stability that are on par with more traditional silicon-based semiconductors remains challenging [7,8,9,10,11,12,13]. Two-dimensional (2D) thienoacenes, such as tetrathienoanthracene (TTA), combine two important strategies toward solving these drawbacks: The incorporation of sulphur atoms and a rigid structure. The presence of sulphur heteroatoms can strengthen electronic communication between molecules due to larger π-orbitals [14,15]. The rigid star-shaped structure of 2D TTAs can encourage these molecules to aggregate into columnar formations, promoting microstructural control over the morphology of the active layer [2,16,17]. Additionally, conjugated structures containing electronegative heteroatoms also possess superior stability towards oxygen and light when compared to their fully-carbon analogues [18,19,20].

There are a few examples in the literature of the synthesis and incorporation of 2D thienoacenes into organic electronic devices [21]; however, there are limited examples of fused thieno[3,2-*b*]thiophene (TT) motifs, despite the fact that these few derivatives rank among the highest performing 2D thienoacenes. Zhang et al. synthesized a small molecule comprised of a dithieno[3,2-*b*:2′,3′-*d*]thiophene core with nine fused rings and seven sulphur atoms [22]. This molecule displayed a forty-fold improvement in charge mobility in OTFTs compared to the parent TTA due to its increased affinity for self-assembly into crystalline nanoribbons in solution. A cyclized thienothiophene core with benzothiophene arms has shown excellent hole mobility in the single-crystal state compared to its shorter thiophene analogue, owing to strong intermolecular contacts and increased effective π-conjugated surface [23]. Finally, a rigid dibenzosexithiophene unit has been copolymerized with bithiophene to afford a wide band-gap polymer for photovoltaic applications, albeit with modest performance due to the disordered polymer network [24]. While OTFTs prepared from these TT-based 2D thienoacenes displayed excellent mobilities, the devices were fabricated from single crystals or nanoribbons using a mask method, which is not easily amenable to large-scale high-throughput fabrication techniques. Achieving similar devices performances from OTFTs produced using simple solution-based methodologies would therefore be advantageous.

Previous work from the Brusso group has focused on the synthesis of derivatives of TTA and their nitrogen-containing counterpart tetrathienoacridine [25,26,27,28]. To improve upon their performance, the conjugation of these systems was extended in two dimensions with oligothienyls of increasing lengths, as it was anticipated that such an approach would enhance the degree of intermolecular communication. Of these derivatives, OTFTs were fabricated using both mono- and bi-thienyl TTA molecules through simple solution-based methodologies. Moderate device performance was observed, with the best mobilities on the order of 10^−4^–10^−3^ cm^2^/Vs. Little improvement in OTFT performance was observed upon expanding the conjugation, likely as a result of increased disorder in the solution-processed thin films. In an effort to overcome this limitation, TT moieties were incorporated into the construction of 2D thienoacenes. The rigid structure of TTs should lead to increased intermolecular interactions in the solid-state, better π-electron delocalization and enhanced light absorptivity and charge conductivity, while simultaneously tuning the energy levels on the molecular orbitals by virtue of the electron-rich nature of the TT core [29,30].

The Brusso group recently reported the synthesis of four novel rigid 2D thienoacenes and thienoacridines that incorporate the TT motif in the arms and/or the core of star-shaped frameworks [31]. Star-shaped architectures were chosen so that the π-framework could be extended in 2D, allowing for the potential for enhanced stability and intramolecular interactions. Here, we report the first successful incorporation of these novel molecules into OTFTs and identify routes to improve device performance through solution process engineering. Unlike previous studies that focused on one compound, this study investigates several derivatives with the TT moiety in different locations.

## 2. Results

**Figure 1:**
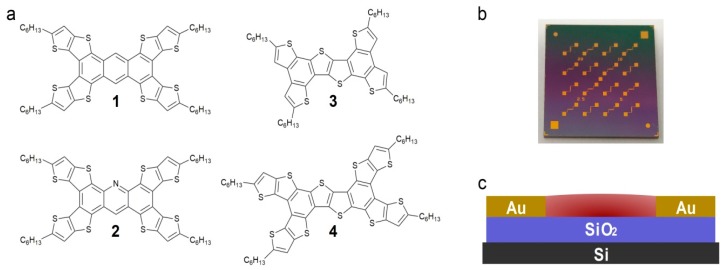
(**a**) molecular structure of the 2D thienoacenes investigated, (**b**) photo of a Fraunhofer chip used and (**c**) the bottom-gate bottom-contact (BGBC) OTFT architecture of the devices on the chip.

Figure 1a depicts the structures of the four TT-containing molecules used in this study. Tetra(5-hexyl)thieno([3,2-*b*]thieno)anthracene (**1**) and tetra(5-hexylthieno[3,2-*b*]thieno)acridine (**2**) contain TT moieties fused to an anthracene or acridine core, respectively. Tetra(5-hexylthieno)benzothieno[3,2-*b*]benzothiophene (**3**) possesses a TT core with thienyl arms, while tetra(5-hexylthieno[3,2-*b*]thieno)benzothieno[3,2-*b*]benzothiophene (**4**) contains both a TT core and four TT arms. All four of the star-shaped molecules are functionalized with four alkyl chains to promote solubility. The synthesis, optical and electrochemical properties of compounds **1**–**4** has been reported previously [31].

Bottom-gate bottom-contact (BGBC) OTFTs (Figure 1b,c) were constructed using pre-fabricated chips purchased from Fraunhofer IPMS (Dresden, Germany). The gate electrode was n-doped Si, with a 230 nm thick SiO_2_ dielectric and Au electrodes. Each chip contained 16 devices (Figure 1b), four sets of four different channel lengths (2.5, 5, 10 and 20 µm); all devices had a channel width of 2000 µm, allowing for a range of W/L ratios to be investigated. Device fabrication involved cleaning the chips with acetone and oxygen plasma before treatment with an octyltrichlorosilane (OTS) toluene solution to form a self-assembled monolayer on the surface of the chips [32]. The organic semiconductor layers were prepared by spin coating a thin film of each of the four thienoacene derivatives onto the chips. All devices were characterized first in vacuum (pressure less than 0.1 Pa) then in air, using the same experimental parameters for both sets of experiments.

**Figure 2:**
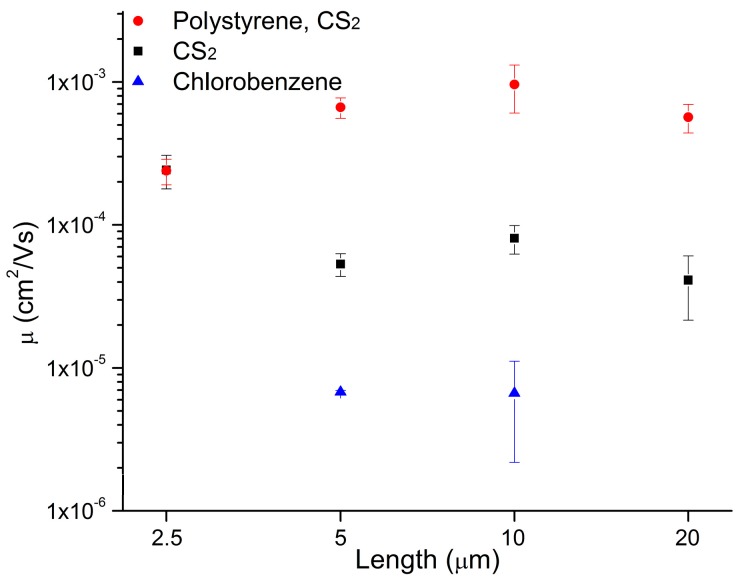
Comparison of average mobilities for 1 devices prepared using chlorobenzene, CS_2_ and a poly(styrene)/CS_2_ blend. All data is for devices tested in air. Working devices were not obtained for the 2.5 and 20 µm channels for the chlorobenzene sample.

The first devices were prepared using chlorobenzene as a solvent, as this was the solvent used in the literature to investigate oligothienyl TTA molecules [26]. The devices prepared with the four TT-containing molecules shown in Figure 1 all demonstrated p-type behavior in both vacuum and air, which is consistent with other reported TT derivatives. The hole mobility versus device channel length is reported in Figure 2. Some of the molecules, in particular **1**, were poorly soluble in chlorobenzene, hindering the formation of a consistent thin film across the entire chip. This inhomogeneity resulted in poor organic semiconductor film quality, which led to low mobilities and inconsistent device performance; this was particularly noticeable for the larger-channel devices (Figure 2). 

Molecules **1**–**4** all displayed good to excellent solubility in CS_2_, which was chosen as the solvent for the next round of experiments. Solutions with concentrations of 5 mg/mL were made for each of the molecules, and devices were prepared again by spin coating. As can be observed in Figure 2 for molecule **1**, hole mobilities increased by an order of magnitude for all channel lengths compared to devices prepared using chlorobenzene as the spin-casting solvent. This increase in device performance was attributed to the enhanced solubility, which allowed for more even film formation. While the performance improvements were impressive, uneven film coverage and pooling was still observed, which is problematic for large area fabrication.

In an effort to further improve device performance, molecules **1**–**4** were each blended with poly(styrene) (PS). Several reports in the literature demonstrate that blending organic semiconductor small molecules with insulating polymers can be an advantageous route towards the fabrication of stable, high-performance OTFTs [33,34]. Blending allows for an amalgamation of the excellent semiconducting properties of the small molecule with the ease of processing and film uniformity afforded by using polymers. The two-component films will phase segregate during the deposition process, with the small molecules accumulating at either the air-thin film or thin film-dielectric interface. Microstructural control of this vertical phase separation has been demonstrated to be dependent on a wide range of processing conditions, including blend ratio, concentrations, solvent, polymer molecular weight and post-thermal treatment. For BGBC OTFT devices, it is essential that the small molecules segregate to the dielectric interface to facilitate efficient charge transport. Yoon and coworkers demonstrated that the utilization of a high-molecular weight polymer allows for the small molecules to accumulate adjacent to the dielectric, whereas a combination of a smaller-molecular weight polymer and thermal annealing promoted segregation of the small molecules to the air-thin film interface [35].

For our experiments, a high-molecular weight PS (M_n_ = 194 kDa) was used to assist in the formation of the semiconducting layer at the dielectric interface. Samples were prepared with each of the thienoacenes such that there was a 1:1 wt/wt ratio of PS to small molecule, with CS_2_ again employed as the solvent. As shown in Figure 2, blending with PS resulted in further improvement in our OTFTs. The hole mobilities increased by an additional order of magnitude for the larger channel devices, corresponding to an increase of over 1000% for the 10 and 20 µm channel lengths. Additionally, it was observed that device variability decreased significantly and that the devices were more robust when tested in air. This increased reliability is likely due to the improved film forming characteristics of the polymer-small molecule blend.

**Figure 3:**
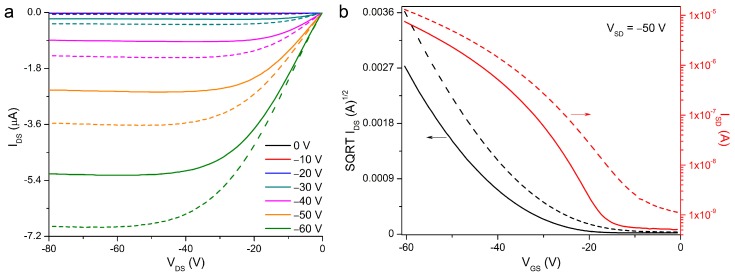
Example (**a**) output and (**b**) transfer curves for **4** blended with poly(styrene) Solid lines represent data obtained in vacuum (P < 0.1 Pa), dashed lines represent data obtained in air. Data in (**b**) is plotted for both the square root (black lines) and log (red lines) of the current.

Example output and transfer curves for the **4**/PS blend in both vacuum and air are shown in Figure 3 for a channel length of 5 µm. The output data in Figure 3a exhibits the typical linear-saturation behavior expected for OTFTs. Device parameters, including both average and highest mobilities (*µ*), on-off ratios (*I_on/off_*) and threshold voltages (*V_T_*) can be found in Table 1 (for a channel length of 5 µm) or in Tables S1–S3 in the Supplementary Materials (for the remaining three channel lengths). 

**Figure 4:**
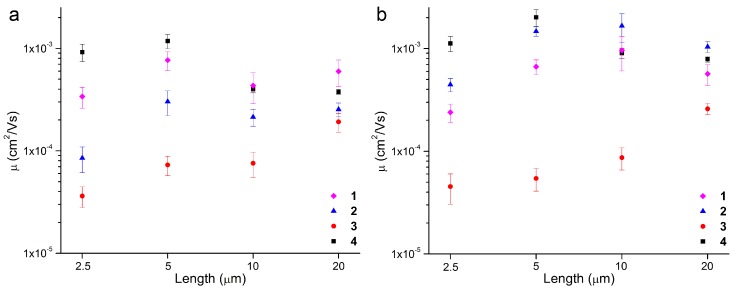
Comparison of mobility data for devices tested in (**a**) vacuum and (**b**) air.

Figure 4 plots the average mobilities for all four thienoacene blends in both air and vacuum with respect to channel length. Interestingly, slightly different trends were observed for the four small molecules when comparing performance in air to vacuum or between the different channel lengths. In general, devices prepared with **4**/PS blend demonstrated superior performance in air compared to vacuum for all channel lengths, with a 20–125% increase in mobility observed. The highest mobility of all the devices tested was obtained for a **4**/PS device in air, with a maximum mobility of 9.2 × 10^−3^ cm^2^/Vs for a channel length of 5 µm. These mobilities are not as high as the values reported for TT-based OTFTs prepared using deposition or nanoribbon mask methodologies, but a direct comparison with our solution-processed devices is not completely valid due to the drastically different semiconducting films that can be obtained. A more logical comparison would be to compare OTFTs prepared with our anthracene and acridine derivatives (molecules **1** and **2,** respectively) with previously reported anthracene molecules. The highest mobility obtained for molecule **1** represents a 1740% increase over previously-reported solution-processable anthracene molecules. For molecule **2**, the highest mobility of 6.2 × 10^−3^ cm^2^/Vs represents a 2380% increase in mobility [28]. 

The hole mobilities of the **1**/PS devices in vacuum and air were relatively equivalent and consistent for the four device channel lengths (on the order of 5 × 10^−4^ cm^2^/Vs). A slightly higher average mobility was achieved in vacuum compared to air for all but the devices with a channel length of 10 μm. The most dramatic change in mobilities when comparing data obtained in vacuum to air occurred for **2**/PS devices. A 400–700% increase in mobility was achieved for the devices when characterized in air, leading to an average mobility of 1.6 × 10^−3^ cm^2^/Vs for the 10 µm channel devices in air. We surmise the enhanced performance for the **2** devices is attributed to the presence of the nitrogen atom in the acridine core, which makes this molecule more stable in air compared to derivatives with an anthracene core [18,19].

Out of the four compounds investigated in this study, devices prepared using **3**/PS were consistently the least performing at all channel lengths, for both vacuum and air experiments. The average mobilities for these devices were two orders of magnitude lower compared to **4**/PS at channel lengths of 2.5 and 5 µm, resulting in a 2300–3600% decrease in average mobility at these two channel lengths (Table 1). Interestingly, the best performance for the **3**/PS devices was achieved at the largest channel length, with average mobilities reaching 2.6 × 10^−4^ cm^2^/Vs in air. This value was still two orders of magnitude lower than the average mobilities for **2**/PS devices (1.3 × 10^−3^ cm^2^/Vs) for the same channel length.

Ranking the OTFT performance of the four thienoacenes in terms of mobilities makes sense when the conjugation length of the four molecules is considered. Molecule **3** has the shortest conjugation path, and, consequently, devices prepared with this molecule consistently underperformed compared to the others, under all studied processing conditions. The conjugation length for **1** is equivalent to **2**; consequently, these two molecules show average mobilities that are roughly on the same order of magnitude in vacuum. However, in air, the increased stability of **2** compared to **1** enables devices containing the former to outperform those prepared with the latter. For the shortest channel lengths, the highest mobilities were obtained for **4**, which also possesses the longest conjugation path. With the larger channels characterized in air, the mobility of the **2**/PS devices surpassed that of the **4**/PS devices. This could be attributed to a favorable doping of **2** in air, or better long-order film morphology for **2** compared to **4**.

Threshold voltages (*V_T_*) for the different molecules varied between −7 and −37 V in vacuum, and between −1 and −21 V in air (Figure S1, Supplementary Materials). However, notable increases in current often occur close to 0 V, with the threshold voltage appearing high in some cases due to deviation from ideal saturation-regime current-voltage behavior Equation (1) (see Supplementary Materials in the measurements). Figure S2 shows how the mobility and *V_T_* of the **4**/PS blend varies for one device as a function of gate voltage. Noticeable charge mobilization begins just below 0 V in air and just below −20 V for vacuum. The presence of oxygen in the air modifies the electronic environment in the film, facilitating hole transport at lower gate voltages without causing an excessive increase in current at 0 V as in some polymers like poly(3-hexylthiophene) (P3HT). Thus, **4** could operate at a relatively low operating voltage in air but not vacuum, despite the apparently high threshold voltage for some samples. On the other hand, devices made with **1** have an average threshold voltage of around −1 V. In air, they demonstrate higher off currents and therefore lower on/off current ratios (*I_on/off_*) of 10^1^ to 10^2^ due to induced charge carriers (doping) at 0 V.

## 3. Conclusions

In conclusion, we have reported for the first time the incorporation of four novel star-shaped 2D thienoacenes, which contain a TT moiety in the core and/or arms of the molecule, into OTFTs. All four organic semiconductors were incorporated into p-type devices and tested in both a vacuum and air. The best field-effect mobilities approached 10^−2^ cm^2^/Vs for devices using **4** or **2** when characterized in air. This represents an increase in performance of 160% over devices with 2D thienoacenes previously reported by the Brusso group. Among the few reports using solution processed molecules containing similar functional groups, our average mobilities are comparable to the best mobilities. Blending the small molecules with poly(styrene) resulted in improved performance as well as improved consistency in devices. The stability and enhanced performance of these molecules means that strict air-free fabrication techniques are not required for device manufacturing. We have therefore identified a robust method for obtaining consistent films and ultimately consistent OTFT characteristics using solution processable TT containing molecules. This novel class of materials is worthy of further investigation, with the possibility of improved device performance through molecular design and device engineering; in particular, improving the solubility of the TT derivatives or substituting the insulating polymer could further improve OTFT performance from this class of molecules. Overall, these results represent a useful starting point for the development of solution-processed devices containing TT moieties.

## Figures and Tables

**Table 1:** Properties of devices tested in vacuum and air for a channel length of 5 µm.

Name	Vacuum				Air			
	Average *µ* (cm^2^/Vs) × 10^−4^	Highest *µ* (cm^2^/Vs)	*I_on/off_*	*V_T_* (*V*)	Average *µ* (cm^2^/Vs) × 10^−4^	Highest *µ* (cm^2^/Vs)	*I_on/off_*	*V_T_ (V)*
1	7.7 ± 1.6	2.3 × 10^−3^	10–10^3^	−8 ± 2	6.7 ± 1.1	1.4 × 10^−3^	10–10^2^	−0.96 ± 4
2	3.0 ± 0.82	2.5 × 10^−3^	10^2^–10^4^	−23 ± 0.9	15 ± 1.5	3.9 × 10^−3^	10^2^–10^3^	−16 ± 0.6
3	0.73 ± 0.16	2.2 × 10^−4^	10–10^3^	−16 ± 2	0.54 ± 0.14	2.4 × 10^−4^	10–10^3^	−13 ± 3
4	12 ± 1.7	3.1 × 10^−3^	10^3^–10^4^	−32 ± 0.6	20 ± 3.8	9.2 × 10^−3^	10^3^–10^4^	−28 ± 1

## Supplementary Materials

The following materials are available online at www.mdpi.com/1996-1944/11/1/8/s1. Tables S1–S3 provides device data for all molecules (tested in vacuum and air) for channel lengths of 2.5, 10 and 20 µm, respectively. Figure S1 compares the average threshold voltages for molecules **1**–**4** in vacuum and air, while Figure S2 depicts device mobility and threshold voltage as a function of gate voltage.
