# Supplementary material for: Organic Thin Film Transistors Incorporating Solution Processable Thieno[3,2-b]thiophene Thienoacenes
Source: Materials (Basel). 2017 Dec 22;11(1):8. doi: 10.3390/ma11010008 (PMC5793506; doi:10.3390/ma11010008)
Supplement: Supplementary file 1 [file materials-11-00008-s001.pdf]

Organic Thin Film Transistors Incorporating Solution Processable Thieno[3,2-*b*]thiophene Thienoacenes

Nicole A. Rice[1], François Magnan[2], Owen A. Melville[1], Jaclyn L. Brusso[2] and Benoît H. Lessard[1]

[1] University of Ottawa, Department of Chemical and Biological Engineering, 161 Louis Pasteur, Ottawa, Ontario, K1N 6N5

[2] University of Ottawa, Department of Chemistry and Biomolecular Science, 10 Marie Curie, Ottawa, Ontario, K1N 6N5

E-mail: benoit.lessard@uottawa.ca (BHL) and jbrusso@uottawa.ca (JLB)

Supporting Materials

Experimental

All solvents and reagents were purchased from commercial sources and used without further purification. The synthesis and purification of the four thienoacenes and thienoacridines has been reported previously [1]. The compounds were further purified by a second recrystallization step before incorporation into devices. Plasma treatment was performed using a Harrick Plasma Cleaner (PDC-32G). Thin films were spin coated using a Laurell Spin Coater, Model WS-650MZ-23NPPB. Electrical measurements were performed on a custom electrical probe station with environmental chamber with controlled atmosphere, oesProbe A10000-P290 (Element Instrumentation Inc. & Kreus Design Inc.) using a Keithley 2614B to set V_{DS} and V_{GS} and to measure I_{DS}. Voltages were increased in a step-wise manner, rather than pulsed, with a delay of 100 ms between measurements. Each device was tested four times. Transfer curves were measured in the saturation regime and were modelled using the following equation:

$$I_{DS} = \frac{\mu C_i W}{2L}(V_{GS} - V_T)^2 \qquad \textbf{Equation 1}$$

where L and W are the channel length and width, respectively. C_i is the capacitance density of the gate dielectric, calculated using $C_i = \frac{\varepsilon_0 \varepsilon_r}{t}$, where t is the thickness of the dielectric (230 nm) and

ε_r is the relative dielectric constant of SiO_2 (3.9). μ is the field-effect mobility and is calculated from the slope of best fit though the most linear region of $\sqrt{I_{DS}}$ plotted against V_{GS}. The threshold voltage V_T is calculated as the x-intercept of the same line fitting.

Bottom-gate bottom-contact devices were constructed using pre-fabricated OTFT chips (Generation 5) purchased from Fraunhofer Institute for Photonic Microsystems (IPMS). The gate was n-doped Si, with a 230 nm SiO_2 dielectric; the drain and source electrodes were Au (30 nm), with a 10 nm ITO adhesion layer. Each chip contained 16 transistors, four each of the four different channel lengths: 2.5, 5, 10 and 20 μm. The width of all the devices was 2000 μm. Device data was averaged for a minimum of 8 devices, with the exception of **2** which experienced some device degradation in air.

Device Preparation

All device preparation was performed in air. The Fraunhofer chips were rinsed well with acetone (to remove the resist) and plasma treated for 15 min. The chips were rinsed with water and isopropanol and dried with N_2 before being submerged in a 1% solution of octyltrichlorosilane (OTS) in toluene. The chips were left sitting in the OTS/toluene mixture for 1 h at 70 °C, before they were rinsed well with toluene and dried in a vacuum oven at 70 °C, again for 1h.

For the devices prepared using chlorobenzene or CS_2 (no poly(styrene)), 5 mg of each molecule was weighed out and dissolved in 1 mL of either chlorobenzene or CS_2. The solutions were heated to either 90 °C (for chlorobenzene) or 60 °C (for CS_2) before being filtered through a 0.2 μm PTFE membrane. Thin films were prepared by static spin coating (500 rpm for 1 min) 200 μL of solution at room temperature in air.

A 5 mg/mL stock solution of poly(styrene) (PS, M_n = 194 kDa) in CS_2 was prepared. 5 mg of each of the four thienoacenes was weighed out into separate vials, and 1 mL of the PS/CS_2 solution was added to each of the vials (final ratio of thienoacene:PS in solution was 1:1 wt/wt). The solutions were heated at 60 °C for 10 minutes, allowed to cool to room temperature and then filtered through PTFE membranes (pore size of 0.2 μm). Thin films were prepared by static spin coating (2500 rpm for one minute) 200 μL of each of the small molecule solutions at room

temperature in air. The chips were dried in a vacuum oven for 1 h at 70 °C, stored under vacuum, and tested within 48 h of fabrication. Testing was carried out in both vacuum (pressure less than 0.1 Pa) and air; testing in vacuum was performed first, followed immediately by testing in air. Devices were allowed to acclimatize for 30 min in both air and vacuum before testing.

Table S1. Device data for all thienoacenes and thienoacridines for a channel length of 2.5 μm.

	Vacuum				Air			
**Name**	**Average μ (cm^2/Vs) x10^{-4}**	**Highest μ (cm^2/Vs)**	**$I_{on/off}$**	**V_T (V)**	**Average μ (cm^2/Vs) x10^{-4}**	**Highest μ (cm^2/Vs)**	**$I_{on/off}$**	**V_T (V)**
**1**	3.4 ± 0.78	1.4 x 10^{-3}	10-10^3	3.6 ± 4	2.4 ± 0.49	9.1 x 10^{-4}	10-10^2	8.8 ± 4
**2**	0.85 ± 0.24	5.5 x 10^{-4}	10-10^4	-2.1 ± 3	4.5 ± 0.67	1.1 x 10^{-3}	10-10^3	-3.2 ± 1
**3**	0.36 ± 0.0082	1.4 x 10^{-4}	10-10^2	5.7 ± 3	0.45 ± 0.15	2.3 x 10^{-4}	10-10^3	0.0078 ± 4
**4**	9.2 ± 1.8	2.3 x 10^{-3}	10^3-10^4	-30 ± 1	11 ± 1.9	2.7 x 10^{-3}	10^3	-25 ± 0.8

Table S2. Device data for all thienoacenes and thienoacridines for a channel length of 10 μm.

	Vacuum				Air			
**Name**	**Average μ (cm^2/Vs) x10^{-4}**	**Highest μ (cm^2/Vs)**	**$I_{on/off}$**	**V_T (V)**	**Average μ (cm^2/Vs) x10^{-4}**	**Highest μ (cm^2/Vs)**	**$I_{on/off}$**	**V_T (V)**
**1**	4.3 ± 1.4	2.5 x 10^{-3}	10-10^4	-15 ± 3	9.6 ± 3.6	4.6 x 10^{-3}	10-10^3	-4.0 ± 5
**2**	2.1 ± 0.40	7.0 x 10^{-4}	10^2-10^3	-26 ± 2	17 ± 5.2	6.2 x 10^{-3}	10^2-10^4	-16 ± 2
**3**	0.76 ± 0.21	3.2 x 10^{-4}	10-10^3	-15 ± 2	0.87 ± 0.21	3.3 x 10^{-4}	10^2-10^3	-8.2 ± 1
**4**	4.0 ± 0.31	6.5 x 10^{-4}	10^2-10^3	-36 ± 0.5	9.0 ± 1.0	1.6 x 10^{-3}	10^3	-29 ± 0.6

Table S3. Device data for all thienoacenes and thienoacridines for a channel length of 20 μm.

	Vacuum				Air			
**Name**	**Average μ (cm^2/Vs) x10^{-4}**	**Highest μ (cm^2/Vs)**	**$I_{on/off}$**	**V_T (V)**	**Average μ (cm^2/Vs) x10^{-4}**	**Highest μ (cm^2/Vs)**	**$I_{on/off}$**	**V_T (V)**
**1**	6.0 ± 1.7	2.2 x 10^{-3}	10^2-10^3	-15 ± 3	5.7 ± 1.3	1.7 x 10^{-3}	10-10^3	-9.5 ± 3
**2**	2.5 ± 0.39	9.4 x 10^{-4}	10^2-10^3	-28 ± 0.8	13 ± 1.3	2.1 x 10^{-3}	10^3-10^4	-19 ± 0.7
**3**	1.9 ± 0.40	5.5 x 10^{-4}	10-10^3	-23 ± 2	2.6 ± 0.33	5.3 x 10^{-4}	10^2-10^3	-13 ± 1
**4**	3.8 ± 0.22	5.8 x 10^{-4}	10^2	-37 ± 0.4	7.9 ± 0.50	1.3 x 10^{-3}	10^3	-31 ± 0.7

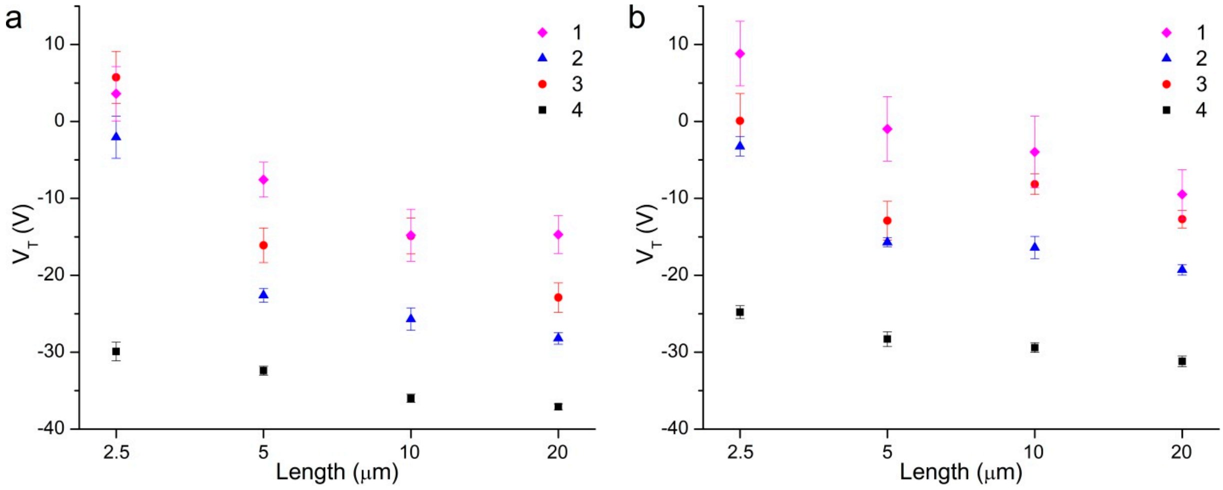

Figure S1. Average threshold voltages for samples tested in (a) vacuum and (b) air.

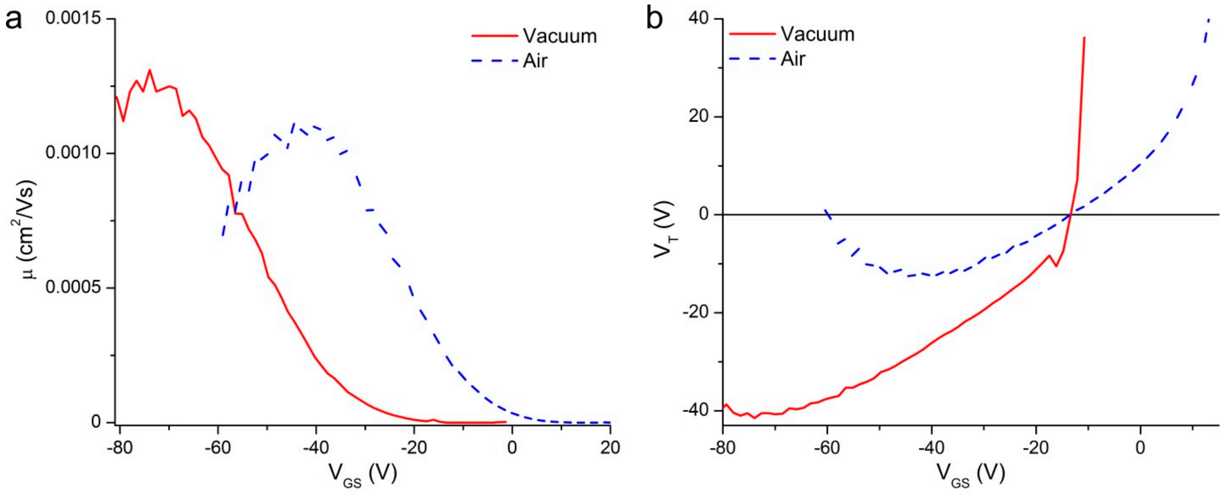

Figure S2. (a) Device mobility and (b) threshold voltage as a function of gate voltage for 4/PS sample (channel length of 5 μm).

References

1. Magnan, F.; Gabidullin, B.; Brusso, J. L. Applying thieno[3,2-b]thiophene as a building block in the design of rigid extended thienoacenes. *RSC Adv.* **2016**, *6*, 97420–97429, doi:10.1039/C6RA18897D.
